# The TOR Pathway at the Neuromuscular Junction: More Than a Metabolic Player?

**DOI:** 10.3389/fnmol.2020.00162

**Published:** 2020-08-28

**Authors:** Perrine Castets, Daniel J. Ham, Markus A. Rüegg

**Affiliations:** ^1^Department of Cell Physiology and Metabolism, Faculty of Medicine, University of Geneva, Geneva, Switzerland; ^2^Biozentrum, University of Basel, Basel, Switzerland

**Keywords:** NMJ, TOR, mTORC1, mTORC2, autophagy, aging, sarcopenia, ALS

## Abstract

The neuromuscular junction (NMJ) is the chemical synapse connecting motor neurons and skeletal muscle fibers. NMJs allow all voluntary movements, and ensure vital functions like breathing. Changes in the structure and function of NMJs are hallmarks of numerous pathological conditions that affect muscle function including sarcopenia, the age-related loss of muscle mass and function. However, the molecular mechanisms leading to the morphological and functional perturbations in the pre- and post-synaptic compartments of the NMJ remain poorly understood. Here, we discuss the role of the metabolic pathway associated to the kinase TOR (*Target of Rapamycin*) in the development, maintenance and alterations of the NMJ. This is of particular interest as the TOR pathway has been implicated in aging, but its role at the NMJ is still ill-defined. We highlight the respective functions of the two TOR-associated complexes, TORC1 and TORC2, and discuss the role of localized protein synthesis and autophagy regulation in motor neuron terminals and sub-synaptic regions of muscle fibers and their possible effects on NMJ maintenance.

## Introduction

Preserving muscle mass and function during aging has emerged as a major public health priority. As populations continue to age in many countries, the societal and personal burden stemming from the natural loss of muscle integrity and thereby life quality of the elderly is growing. The age-related loss of muscle mass (atrophy) and force (weakness), referred to as sarcopenia, is a major contributor to frailty, morbidity and mortality (for a review, see Cruz-Jentoft et al., 2019). Moreover, as frailty and disability increase, reduced activity or even disuse accelerate and aggravate the loss of muscle mass, resulting in a vicious cycle leading to a precipitous decline of the individual (Rezus et al., 2020). The situation is even more dramatic when the geriatric syndrome associates with other pathologic conditions, such as obesity. It is estimated that more than 200 million individuals worldwide will be afflicted by sarcopenia by 2050, with dramatic socioeconomic and clinical implications (Janssen et al., 2004; Cruz-Jentoft et al., 2010; Dhillon and Hasni, 2017). A well-described feature of sarcopenia is the structural changes of neuromuscular junctions (NMJs) (for a review, see Ham and Rüegg, 2018 and Rudolf et al., 2014). NMJs are the synapses connecting motor neurons to muscle fibers. Deterioration of muscle innervation during aging has been documented in several species, and treatments that slow sarcopenia also preserve NMJ integrity (Valdez et al., 2010). Such observations led to the idea that NMJ perturbations are crucial determinants of the initiation and progression of sarcopenia, although the mechanisms responsible for age-dependent NMJ destabilization remain elusive.

**FIGURE 1:**
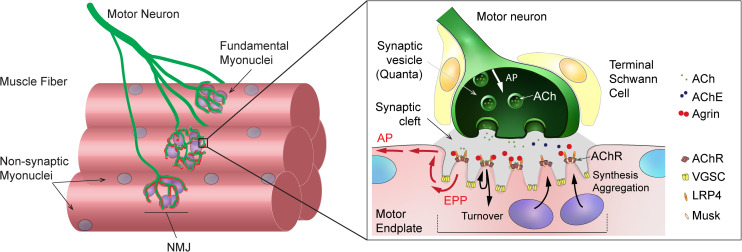
General organization and function of mammalian neuromuscular junction (NMJ). **Left:** Scheme showing a motor axon, which branches and forms NMJs to innervate skeletal muscle fibers. Sub-synaptic (or fundamental) nuclei are seen underneath the NMJ. **Right:** Enlarged view of NMJ, showing capping Schwann cells in close contact with the muscle and axon, the junctional folds and the accumulation of synaptic proteins at the post-synaptic muscle membrane, and the liberation of synaptic vesicles at the pre-synaptic membrane upon action potential arrival. ACh, acetylcholine; AChE, acetylcholinesterase; AChR, acetylcholine receptor; AP, action potential; EPP, endplate potential; LRP4, LDL Receptor Related Protein 4; VGSC, voltage-gated sodium channel.

Neuromuscular junctions are highly specialized chemical synapses, designed to transmit action potentials from pre-synaptic motor neurons to post-synaptic muscle fibers, thereby initiating muscle contraction. The formation and maintenance of NMJs require a complex interplay between nerves, muscle fibers and terminal Schwann cells (for a review, see Tintignac et al., 2015). In muscle fibers, post-synaptic proteins specifically accumulate at the sarcolemma immediately beneath the contact point between nerve and muscle, ultimately leading to motor endplate formation (Figure 1). In adult, innervated muscle, transcription of synaptic genes, encoding post-synaptic proteins, is confined to sub-synaptic (also called fundamental) myonuclei and repressed in all non-synaptic myonuclei. Previous work has uncovered several epigenetic and transcriptional effectors that ensure the tight constraint of synaptic gene expression to fundamental myonuclei. Similarly, various factors have been implicated in regulating synaptic protein dynamics (e.g., acetylcholine receptor clustering and internalization) at the motor endplate (for a review, see Li et al., 2018). A complex network of effectors and compartments is crucial for NMJ maintenance and, hence, NMJ destabilization as a consequence of a disease or aging can be initiated by pre-synaptic motor neurons, terminal Schwann cells or muscle fibers (Li et al., 2018, 2019; Martineau et al., 2018).

One of the underlying drivers of the aging process is a change in proteostasis, the balance between protein synthesis and protein degradation (Tan et al., 2019). Alterations in proteostasis have also been implicated in neurodegenerative diseases, such as Amyotrophic Lateral Sclerosis (ALS) (Ferri and Coccurello, 2017). TOR (*Target Of Rapamycin*) is at the heart of one of the key signaling pathways responsible for proteostasis. TOR activity is essential for muscle development and growth, however, overactive TOR signaling is also implicated in aging and sarcopenia (Tang et al., 2019; Ham et al.,in press) and suppressing TOR by pharmacological or nutritional means remains a primary target of anti-aging interventions. While TOR is well known for its role in metabolism and aging, its role in NMJ development and maintenance is less well described. Given the broad medical interest in TOR activity manipulation and the essential role of the NMJ in maintaining skeletal muscle function and thereby mobility, it is important to establish the impact and importance of TOR activity on the NMJ. In this review, we discuss the role of TOR on NMJ stability and function in different species and compartments, and examine the proposed underlying mechanisms linking TOR to NMJ maintenance.

## The TOR Signaling Network

### TOR and TOR-Associated Proteins

**FIGURE 2:**
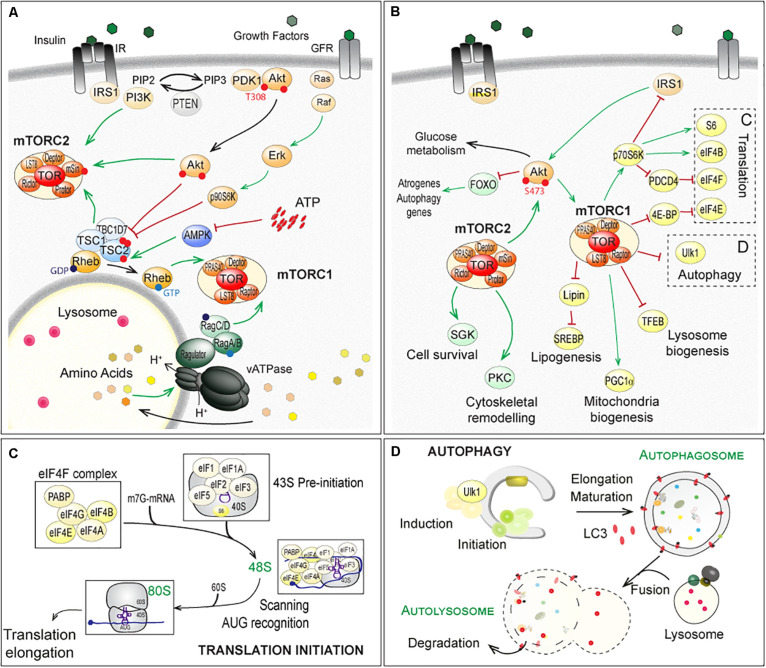
Regulation of (m)TOR pathways and downstream factors and processes in fly and mammals. **(A)** Up-stream mechanisms regulating the (m)TOR-associated complex (m)TORC1 and (m)TORC2. Extracellular stimuli, nutrients and energy contribute to the regulation of mTORC1, at the lysosome. Growth factors, such as insulin, activate PKB/Akt, *via* the recruitment of IRS1 and PI3K and the formation of PIP3 (*PhosphatidylInositide-3,4,5-triPhosphate*). Active PKB/Akt destabilizes the complex formed by TSC1, TSC2 and TBC1D7, which releases Rheb and in turn activates (m)TORC1. Similarly, ERKs (*Extracellular signal-Regulated Kinases*) and p90S6K (*p90 ribosomal S6Kinase*, also known as *MAPK-activated protein kinase-1*) phosphorylate TSC2 and activate (m)TORC1, in response to growth factors (Ma et al., 2005; Roux et al., 2007). Amino acids activate the vacuolar proton pomp v-ATPase, which acidifies lysosome and promotes GTP or GDP loading on Rag proteins. Interaction of Raptor with GTP-bound RagA/B and GDP-bound RagC/D leads to the activation of (m)TORC1 after its recruitment at the lysosomal membrane (Kim et al., 2008; Sancak et al., 2010; Zoncu et al., 2011; Bar-Peled et al., 2012). AMPK (*AMP-activated Kinase*) inhibits (m)TORC1 by phosphorylating Raptor (Gwinn et al., 2008) and TSC2 (Inoki et al., 2006), in response to a lowering of cellular energy (*i.e.* AMP/ATP levels). PI3K, PKB/Akt and TSC1/TSC2 complex activate mTORC2. **(B–D)** Downstream effectors of (m)TORC1 and (m)TORC2, with special focus on translation initiation **(C)** and autophagy **(D)**, both mainly controlled by (m)TORC1. Note that mTORC1 indirectly inhibits PKB/Akt and mTORC2, *via* p70S6K and the degradation of IRS1. In contrast, mTORC2 promotes mTORC1, by activating PKB/Akt. Red lines indicate inhibition; Green arrows represent activation. TFEB, Transcription factor EB; SREBP, Sterol regulatory element-binding proteins; PGC1α, Peroxisome proliferator-activated receptor gamma coactivator 1-alpha. The other abbreviations are included in the text.

Target of Rapamycin is a serine/threonine kinase, fortuitously identified in yeast mutants based on their resistance to the antifungal and immunosuppressive drug rapamycin (Heitman et al., 1991; Loewith et al., 2002). Since its identification in yeast, homologs of TOR have been identified in all eukaryotes. In the following, we will limit the discussion to **(m)TOR,** referring to mammals (mTOR for *mammalian* or *mechanistic TOR*) or flies (TOR), as most reports regarding NMJ physiology have been obtained in human, rodents and *Drosophila*. Although mammals and flies possess only one *(m)TOR* gene, the (m)TOR protein assembles into two distinct multi-protein complexes, (m)TORC1 and (m)TORC2 (Soulard et al., 2009; Gonzalez and Hall, 2017; Kim and Guan, 2019). Raptor (*Regulatory associated-protein of mTOR*) and PRAS40 (*40kDa Proline-Rich Akt Substrate*) specifically complement (m)TORC1, while (m)TORC2 comprises Rictor (*Rapamycin-insensitive companion of mTOR*), mSin1 (*mammalian stress-activated protein kinase-interacting protein 1*) and Protor1/2 (*Protein observed with rictor*) (Figure 2A). Although originally considered mTORC1 specific, several reports now show that long-term exposure to rapamycin also dampens mTORC2 activity (Lamming et al., 2012).

### Regulation and Subcellular Localization of (m)TOR Complexes

(m)TORC1 is a central sensor of extra- and intra-cellular stimuli, including growth factors, amino acids and energy (for a detailed review, see Gonzalez et al., 2020). Growth factors, such as IGF-1 (*Insulin-like Growth Factor 1*), activate PKB/Akt (*Protein Kinase B*/*Akt*), *via* the recruitment of IRS1 (*Insulin Receptor Substrate 1*) and PI3K (*PhosphoInositide-3-Kinases*) (Figure 2A). Active PKB/Akt phosphorylates TSC2 (*Tuberosis Sclerosis Complex 2*), and thereby destabilizes the complex formed by TSC1, TSC2 and TBC1D7 (*TBC1Domain family member 7*) (Inoki et al., 2002; Manning et al., 2002; Potter et al., 2002). This releases the small, lysosomal GTP-binding protein Rheb (*Ras Homolog Enriched in Brain*), which in turn activates (m)TORC1 (Inoki et al., 2003a,b; Tee et al., 2003; Long et al., 2005). Levels of free amino acids regulate (m)TORC1 activation, *via* the family of Rag GTPases (*small Guanosine TriPhosphatases*) and Ragulator (Kim et al., 2008; Sancak et al., 2010; Zoncu et al., 2011; Bar-Peled et al., 2012; Figure 2A).

Signaling up- and downstream of mTORC2 is less well described. PKB/Akt is known to activate mTORC2 by phosphorylating mSin1 (Zinzalla et al., 2011; Yang et al., 2015) and in turn, (m)TORC2 phosphorylates and activates PKB/Akt by phosphorylating Ser473 (Jacinto et al., 2006). Paradoxically, the TSC1/TSC2 complex, while inhibiting mTORC1, activates mTORC2 independently from Rheb (Yang et al., 2006; Huang et al., 2008, 2009; Figure 2A). By controlling p70S6K (*p70 ribosomal S6Kinase*, or S6K), which targets the degradation of IRS1, mTORC1 indirectly inhibits PKB/Akt and mTORC2 (Haruta et al., 2000; Manning et al., 2005). By inducing PKB/Akt, mTORC2 promotes mTORC1. These intricate regulations and feedbacks make it difficult to identify the primary cause for the deregulation of (m)TOR targets.

### (m)TOR Targets Accumulate at the NMJ

(m)TORC1 promotes cell growth, by activating anabolic processes (*e.g.* protein or lipid synthesis) and by inihibiting catabolic processes, such as autophagy (Figures 2A–D). The main direct targets of (m)TORC1, 4E-BP1 (*eIF4E-Binding Protein 1*) and S6Ks are involved in the regulation of cap-dependent translation (Figure 2B). (m)TORC1-dependent activation of S6Ks triggers phosphorylation of several targets, including the S6 ribosomal protein, while (m)TORC1-dependent phosphorylation of 4E-BP1 releases inhibition of the translation factor eIF4E (*eukaryotic translation Initiation Factor 4E*). The best characterized targets of mTORC2 include SGK1 (*Serum and Glucocorticoid-regulated Kinase 1*), PKCs (*Protein Kinases C*) and PKB/Akt, which mediate the effects of mTORC2 on cell survival and cell migration (Figure 2B).

While (m)TOR itself has not been localized to NMJs, several (m)TORC1 targets including eIF4E in *Drosophila* larvae (Sigrist et al., 2000) and phosphorylated forms of S6 in mice (Tang et al., 2014; Castets et al., 2019) are enriched at NMJs. mTORC1 activity is also strongly regulated by innervation status, with experimental denervation potently stimulating mTORC1 activity in fast-type mouse skeletal muscle (Quy et al., 2013; Tang et al., 2014; Castets et al., 2019). Transient activation of mTORC1 has also been observed under other muscle wasting (catabolic) conditions that reduce neural stimulation, such as hindlimb suspension, where mTORC1 activity peaks after 6 h and returns to basal levels after 12 h (Chibalin et al., 2018). These observations point to (m)TOR as an early sensor of lost innervation in skeletal muscle. Activation of mTORC1 in situations of muscle wasting seems paradoxical as mTORC1 is also activated during muscle growth. However, as free amino acids are both necessary and sufficient to activate mTORC1, it is conceivable that this counter-intuitive increase in mTORC1 activity during catabolic conditions results from the release of amino acids by muscle protein breakdown. While future work is still required to determine whether mTORC1 is helpful or harmful during muscle atrophy (Quy et al., 2013; Tang et al., 2014; Castets et al., 2019), the enrichment of TOR targets at the NMJ and the marked increase in TOR activity following lost innervation points to a prominent role of TOR in NMJ physiology.

## Consequences of (M)TOR Deregulation on NMJ Development and Maintenance: Lessons From Animal Models

The importance of the (m)TOR pathway in the central nervous system is highlighted by the Tuberous Sclerosis syndrome caused by mutations in the *TSC1* or *TSC2* genes, which result in benign tumors associated with neurological anomalies. Mutations of *TSC1/2* alter brain development and synaptic function (for a review, see Polchi et al., 2018). There is also evidence that changes in TSC-TOR signaling affect the peripheral nervous system, in particular the development and function of NMJs. Modern techniques have allowed extensive characterization of the consequences of specific manipulations of the (m)TOR pathway in both pre- and post-synaptic compartments on the structure and the function of NMJs.

### NMJ Development and Maintenance in *Drosophila* With TOR Deregulation

**FIGURE 3:**
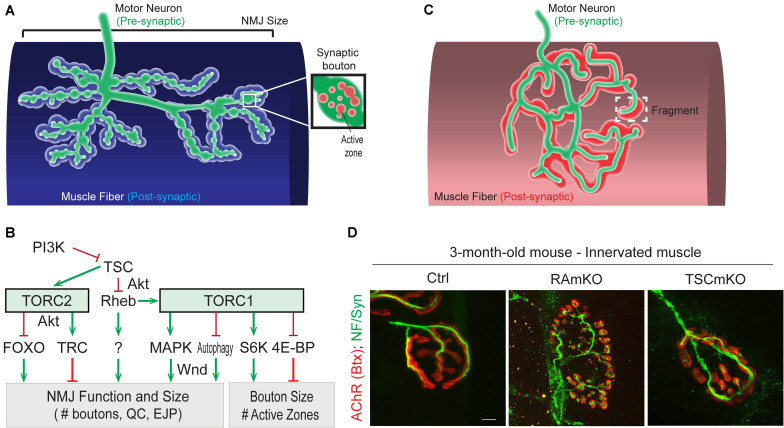
Role of (m)TORC1 signaling in NMJ maintenance. **(A)** NMJ organization in *Drosophila*, showing the synaptic boutons spreading on the muscle fiber. **(B)** TORC1 and TORC2 signaling regulate the size of the NMJ (number of synaptic boutons), via the MAPK pathway, the autophagy process, FOXOs and the kinase Trc. TORC1 also controls the size of the synaptic boutons, via S6K and 4E-BP1. **(C,D)** Typical pretzel-shape structure of rodent NMJ (up), with the pre-synaptic compartment (motor axon) shown in green and the post-synaptic region in red. Deregulation of mTORC1 in muscle leads to NMJ alterations, with an increased fragmentation of the motor endplate (D). Motor axon is stained with Neurofilament and Synaptotagmin (green); AChRs at the muscle membrane are labeled with fluorescent bungarotoxin (red). Scale bar, 10 μm.

*Drosophila* NMJs, especially at the third larval stage, are a commonly used model system to study the role of specific genes in synapse development and plasticity. One major difference to vertebrate NMJs is that they are glutamatergic synapses, which has been used to argue that they are more suitable as a model for synapses of the central nervous system. Moreover, the synaptic cleft of *Drosophila* NMJs is not filled with basal lamina, a highly structured assembly of extracellular matrix proteins. However, thanks to the availability of genetic tools, the accessibility and stereotyped organization of NMJs, and gene homology with vertebrates, studies in *Drosophila* have led to important insights for the NMJ field. In *Drosophila*, the motor neuron establishes a chain of 20-50 synaptic boutons with muscle fibers. Each synaptic bouton contains around ten active zones in the pre-synaptic compartment, each of them often being referred to as a “synapse” (Figure 3A). This characteristic allows the effect of gene mutations on the strength of the connection between motor neuron and muscle to be assessed by simply counting the number of synaptic boutons (for a review, see Collins and DiAntonio, 2007). An overview of *Drosophila* TOR-pathway mutants and their phenotypes is provided in Table 1.

**TABLE 1:** Modulation of the TOR pathway in *Drosophila* larvae and its effects on NMJ.

**Genetic change**	**Pre/Post**	**Morphological changes**	**Electrophysiology**	**References**
*Pi3k* overexpression	MN	Overgrowth	Increased mEPP frequency and amplitude	Martin-Pena et al., 2006; Knox et al., 2007
*Pi3k* DN	MN	Reduced size		Martin-Pena et al., 2006
*Akt1* mutant	Larvae	Overgrowth		Natarajan et al., 2013
	Larvae	Reduced size		Martin-Pena et al., 2006
*Akt* overexpression	MN	Overgrowth		Martin-Pena et al., 2006
*Rheb* overexpression	MN	Overgrowth	Increased QC and EPP	Knox et al., 2007; Natarajan et al., 2013
	Muscle	Normal growth		Knox et al., 2007
*Rheb* mutants	Larvae MN	Reduced size	Reduced mEPP frequency, EPP amplitude and QC	Knox et al., 2007
*Tsc1* mutants	Larvae	Overgrowth		Natarajan et al., 2013
*Tsc2* mutants	Larvae	Overgrowth Increased bouton size	Unchanged EPP and mEPP	Natarajan et al., 2013
*Tsc2* RNAi	MN	Overgrowth		Natarajan et al., 2013
	Muscle	Slight overgrowth		Natarajan et al., 2013
*Tsc1/2* overexpression	MN	Reduced size		Knox et al., 2007
*Rictor* mutant	Larvae	Overgrowth		Natarajan et al., 2013
*Raptor* RNAi	MN	Normal growth Decreased boutons size		Natarajan et al., 2013
		Reduced size		Wong et al., 2015
S6K active form	MN	Overgrowth		Howlett et al., 2008
		Normal growth		Shen and Ganetzky, 2009
*S6k* DN	MN	Normal growth		Shen and Ganetzky, 2009; Wong et al., 2015
*S6k* null mutation	MN	Normal growth Decreased bouton size and AZ number	Decreased EPP amplitude, QC, and mEPP frequency	Knox et al., 2007; Cheng et al., 2011
*Foxo* overexpression	MN	Overgrowth		Nechipurenko and Broihier, 2012
		Normal growth		Howlett et al., 2008
*Trc* mutants	Larvae	Overgrowth		Natarajan et al., 2015
*RagA/C* mutants	Larvae	Reduced size		Wong et al., 2015

*Overgrowth/Reduced size refer to NMJ size, i.e., increased vs. reduced number of synaptic boutons per muscle area. DN, dominant negative. MN, Motoneuron. AZ, Active Zones. QC, Quantal Content (number of synaptic vesicles released upon neuron impulse). EPP, excitatory post-synaptic potential (induced upon neuron stimulation). mEPP, miniature EPP, corresponding to the post-synaptic response to spontaneous release of a single synaptic vesicle.*

While altered post-synaptic TOR signaling has little effect on the size and function of NMJs in *Drosophila*, activation of TOR, *via* the PI3K/TSC/Rheb branch, in motor neurons leads to overgrown NMJs (Martin-Pena et al., 2006; Knox et al., 2007; Natarajan et al., 2013). Inversely, inhibition of TOR, *via* overexpression of *Tsc1/2* or depletion of Rheb reduce NMJ size (Knox et al., 2007), thereby highlighting the essential role of pre-synaptic PI3K/TSC/Rheb/TOR signaling in NMJ development and growth. However, conclusions resulting from experiments focusing on TOR-associated complexes or on downstream targets that potentially mediate these NMJ phenotypes are controversial. Especially, deletion of raptor decreases NMJ size in some cases (Wong et al., 2015), but not in others (Natarajan et al., 2013). On the other hand, most reports conclude that TORC1/S6K promotes growth of synaptic boutons (Cheng et al., 2011; Natarajan et al., 2013), but does not affect their number (Knox et al., 2007; Shen and Ganetzky, 2009; Cheng et al., 2011; Natarajan et al., 2013; Wong et al., 2015). S6K also contributes to the number of active zones at the NMJ (Cheng et al., 2011). Hence, the TORC1/S6K axis regulates structural and functional properties of NMJs in *Drosophila*, but may not mediate the effect on NMJ size (i.e., synaptic bouton number) ascribed to PI3K/TSC/Rheb signaling. A recent study instead indicated that the lysosome-Rag/TORC1 branch promotes NMJ growth *via* the MAPK (*Mitogen-activated protein kinases)/*JNK (*c-Jun N-terminal kinases*) pathway (Wong et al., 2015). Hence, TORC1 in the pre-synaptic compartment would both control the number and the size of synaptic boutons, by regulating MAPK and S6K signaling, respectively (Figure 3B).

Besides the effects of TORC1 activity, several pieces of evidence point to a role of TORC2 in mediating the effects of PI3K/TSC on NMJ growth. TSC activates TORC2, and inhibition of TORC2 by deleting rictor, triggers NMJ overgrowth (Natarajan et al., 2013). The negative effect of TSC/TORC2 on NMJ size could involve the activation of two kinases, PKB/Akt and Trc (*Serine/threonine-protein kinase tricorner*), and the consequent inhibition of FOXO (*Forkhead BoxO*) signaling (Nechipurenko and Broihier, 2012) and WASP proteins, respectively (Natarajan et al., 2015; Figure 3B). Such an inhibitory effect of TORC2/Akt on NMJ size may explain why only high doses of rapamycin (i.e., sufficient to inhibit mTORC2) induce an increase in NMJ size in *Drosophila* larvae (Knox et al., 2007; Shen and Ganetzky, 2009; Wong et al., 2015). However, the role of PKB/Akt remains unclear as PKB/Akt activation also triggers NMJ overgrowth (Natarajan et al., 2013). Further investigation of the role of PKB/Akt in controlling NMJ size are hence required to understand these discrepancies. Similarly, the primary effect of Rheb on synapse size is independent of both TORC1 (Knox et al., 2007) and TORC2 (Natarajan et al., 2013), indicating the existence of alternative downstream effectors.

### Consequences of Suppressing mTOR Signaling on NMJ Development and Maintenance in Mouse

In rodents, NMJs typically display a pretzel-like organization, marked by dense synaptic protein aggregation, including acetylcholine receptors (AChR), in post-synaptic regions (Figure 3C). Fragmentation of the endplate structure into several clusters (detected by staining AChRs with α-bungarotoxin), as well as partial or complete loss of innervation of the endplate region, are common readouts of NMJ health. Table 2 provides an overview of mouse mTORC1 pathway mutants and their corresponding NMJ defects.

**TABLE 2:** Modulation of TOR pathway in mouse and its effects on NMJ.

**Model**	**Effect on signaling pathway**	**Defect/Loss of innervation**	**Changes in post-synaptic compartment**	**Response to nerve injury**	**References**
Inducible mTOR k.o. mTOR depletion	↓ mTORC1/2 (*HSA* promoter)	4% denervated fibers			Baraldo et al., 2020
RAmyfKO Raptor depletion	↓ mTORC1 (*Myf5* promoter)	Abnormal innervation of the diaphragm muscle			Rion et al., 2019a
RAmKO Raptor depletion	↓ mTORC1 (*HSA* promoter)		Extra-synaptic clusters Endplate fragmentation		Bentzinger et al., 2008
Inducible Raptor k.o. Raptor depletion	↓ mTORC1 (*HSA* promoter)	5% denervated fibers (7M) Pre-synaptic changes (7M)	Endplate fragmentation		Baraldo et al., 2020
RImyfKO Rictor depletion	↓ mTORC2 (*Myf5* promoter)	Not observed	Not observed		Rion et al., 2019b
RImKO Rictor depletion	↓ mTORC1 (*HSA* promoter)	Not observed	Not observed		Bentzinger et al., 2008
TSCmKO TSC1 depletion	↑ mTORC1 (*HSA* promoter)	Altered transmission (9M)	Fragmentation (3M) ↓ AChR density (9M)	Endplate degeneration	Castets et al., 2019; Ham et al.,in press

*HSA, Human Skeletal Actin; M, month.*

#### Elimination of mTORC1 Signaling in Muscle

Recent insights into the importance of mTOR in NMJ maintenance come from inducible, muscle-specific mTOR deficient (mTORmKO) mice, where 1 month of mTOR deletion leads to denervated fibers (around 5%) and rapamycin further exacerbates denervation (Zhang et al., 2019; Baraldo et al., 2020). Unfortunately, NMJ phenotypes have not been investigated in the constitutive, severely myopathic mTORmKO mice or mTORmKOKI mice, in which a kinase-dead version of mTOR is expressed in muscle fibers of mTORKO mice and exacerbates the myopathy (Risson et al., 2009; Zhang et al., 2019). Evidence for mTORC1 mediating the effect of mTOR on NMJs came from mTORC1-specific mouse models.

Conditional depletion of Raptor in muscle precursor cells (RAmyfKO mice) during development results in abnormal development of NMJs with aberrant innervation in the diaphragm at embryonic day 17.5 (E17.5) and a severe phenotype with perinatal lethality related to respiratory failure (Rion et al., 2019a). However, it remains unknown if the impaired innervation observed in this mouse model arises directly from a lack of mTORC1 signaling at the NMJ, or from an overall defect in muscle development. Mouse models with mTORC1 depletion in muscle fibers, triggered by constitutive or inducible Raptor depletion, have also been widely analyzed. The first report in 2008 established that RAmKO (*Raptor muscle-specific KO*) mice develop an early, severe myopathy that results in early death at the age of 5-7 months (Bentzinger et al., 2008). There were no gross alterations of NMJs in diaphragm from 3-month-old RAmKO mice, although clusters of AChRs were also found in extra-synaptic regions of muscle fibers, indicative of denervation (Bentzinger et al., 2008). Examination of NMJs in hindlimb muscle from 3-month-old RAmKO mice also revealed an increased fragmentation of NMJs (Castets and Rüegg, unpublished observation) (Figures 3C,D). Although only a small fraction of fibers (1 to 4%) have centralized myonuclei, we cannot rule out that the NMJ phenotype results from muscle fiber degeneration/regeneration events. Similar changes in NMJs have been described in *mdx* mice, a mouse model for Duchenne muscular dystrophy, where degeneration/regeneration is a prominent feature (Haddix et al., 2018). Generation of inducible, muscle-specific Raptor-KO mice offer an opportunity to detect NMJ dysfunction before myopathic onset (Baraldo et al., 2020; Ham et al., 2020). Baraldo et al., reported that one-month Raptor depletion increases the proportion of denervated fibers, which further increases when mice are treated with rapamycin (the idea here being that rapamycin inhibits some residual mTORC1 activity, but non-muscle effects of rapamycin-induced mTORC1 suppression could also contribute). However, even 5 months post-depletion, there was no change in muscle mass, fiber size distribution and *ex vivo* muscle force, suggesting a minor contribution of the muscle denervation to the overall health (Ham et al., 2020). At 7 months post-deletion, the proportion of denervated fibers and the fragmentation of the motor endplate were increased; alterations in the motor neurons were also detected (Baraldo et al., 2020). These results suggest that mTORC1 depletion in muscle fibers affects both post- and pre-synaptic compartments. However, as inducible raptor KO mice also display centro-nucleated fibers at this late stage, one still cannot rule out an influence of muscle degeneration on NMJ alterations. Hence, although mouse models generated in the last decade point to a role of mTORC1 in the development and maintenance of NMJs, its local effect at the NMJ, independent from its function in myofibers, requires further investigations.

#### Depletion of mTORC2 in Muscle

mTORC2 suppression *via* Rictor depletion in muscle precursors (RImyfKO) or muscle fibers (RImKO) show little phenotype (Bentzinger et al., 2008; Kumar et al., 2008; Rion et al., 2019a). Further investigations on NMJ organization and function in these models are required to confirm or oppose a role of mTORC2 in NMJ development and/or maintenance.

### Effects of mTOR Activation in Mouse Muscle on NMJ Maintenance

So far, the consequence of mTORC1 or mTORC2 activation in motor neurons on NMJs has not been studied. Similarly, there is no report assessing the effect of mTORC2 activation in muscle fibers on NMJs in rodents. In contrast, mouse models with constant activation of mTORC1 have been obtained by deleting *Tsc1* specifically in skeletal muscle (TSCmKO mice). TSCmKO mice develop a late-onset myopathy, associated with a progressive accumulation of aggregates, vacuoles and abnormal organelles in muscle fibers, related to autophagy blockade (Castets et al., 2013). At 3 months of age, TSCmKO mice display increased fragmentation of the motor endplate (Figure 3D), with no sign of fiber degeneration, suggesting that constant activation of mTORC1 in muscle is deleterious for NMJ maintenance. Consistently, fragmentation of the motor endplate is exacerbated in 9-month-old TSCmKO mice, together with a reduction in post-synaptic AChR density and impaired transmission properties (Ham et al., in press). Interestingly, nerve sprouting and quantal content are increased at this age, reflecting changes in the pre-synaptic compartment. As in aging (see below), these changes may compensate for the lower AChR density. Increased transmission fatigue and reduction in nerve-induced muscle force confirm the functional impairment of NMJs in TSCmKO mice (Ham et al., in press). Of note, nerve injury dramatically exacerbates the defects observed in TSCmKO muscle, with rapid motor endplate degeneration. In parallel to loss of the original post-synaptic structures, abnormal, enlarged extra-synaptic clusters accumulate throughout muscle fibers (Castets et al., 2019). It remains unclear how these clusters form in non-synaptic regions in spite of failed up-regulation of synaptic genes in response to nerve injury. Whether these clusters represent nascent clusters or derive from degenerating motor endplates remains to be explored. These studies point to a major role of mTORC1 in the maintenance of the NMJ, especially upon challenging conditions, such as nerve injury. NMJ destabilization in response to mTORC1 activation may involve local changes in protein synthesis, autophagy and/or gene expression, as discussed below.

Together, genetic manipulations in *Drosophila* and mouse models highlight the importance of tightly coordinated TOR signaling within both pre- and post-synaptic compartments for NMJ development and maintenance, however, specific aspects of our knowledge is lacking in each organism. While experiments point to a primary role of pre-synaptic TOR signaling in *Drosophila* larvae, studies in adult flies are lacking. In contrast, post-synaptic deregulation of mTORC1 disturbs the NMJ in mice, but these observations cannot yet be untangled from muscle degeneration/regeneration ongoing in some of the mouse models.

## TOR-Dependent Mechanisms Involved in NMJ Maintenance

### (m)TOR and Local Translation in Pre- and Post-synaptic Regions

#### Translation Regulation in Brief

Protein synthesis involves translation initiation, elongation, termination and ribosome recycling. Translation is mainly regulated at the initiation stage by numerous initiation factors (IFs – eIFs for eukaryotic). In cap-dependent translation, initiation requires the interaction of the 5’ capped region (m7GpppN) of mRNAs with the cap-binding complex eIF4F, which includes eIF4E and its partners eIF4G and eIF4A. The ternary structure formed allows binding of the pre-initiation complex containing the 40S ribosomal unit, methionine transfer RNA and eIF1/1A/2/3/5 (Figure 2C). Upon scanning and identification of the AUG initiation codon, the release of eIFs promotes formation of the 80S structure, by recruitment of the 60S subunit, which ensures peptide elongation (for a review, see Sonenberg and Hinnebusch, 2009).

(m)TORC1 promotes protein synthesis by regulating S6K and 4E-BP1 (Figures 2B,C). (m)TORC1 phosphorylates and thereby activates S6K at Thr389, which in turn phosphorylates eIF4B, PDCD4 (*ProgrammeDCell Death protein 4*) and S6 proteins. Phosphorylation of eIF4B increases its interaction with eIF4A in the cap-binding complex, eIF3 in the preinitiation complex (Holz et al., 2005) and the elongation factor eEF2K (Wang et al., 2001). Phosphorylation of PDCD4 releases its inhibitory activity onto eIF4A and thereby promotes formation of the cap-binding eIF4F complex (Yang et al., 2003; Dorrello et al., 2006). *Via* S6, S6K promotes the translation of mRNA with oligopyrimidine tracts (5’TOP), which encode proteins of the translation machinery (Jefferies et al., 1994; Terada et al., 1994). In parallel, 4E-BP1 is a key repressor of translation initiation, by trapping the rate-limiting factor eIF4E of the cap-binding complex. Phosphorylation of 4E-BP1 by mTORC1 allows the dissociation of eIF4E, which binds to eIF4G and initiates cap-dependent translation (Heesom and Denton, 1999; Marcotrigiano et al., 1999). Interaction of eIF4E with PABP (*Poly(A)-Binding Protein*) also facilitates translation (re)-initiation by binding the 3’ end of mRNAs and ensuring the formation of loop structures.

#### Role of Local Translation in Motor Neurons

**FIGURE 4:**
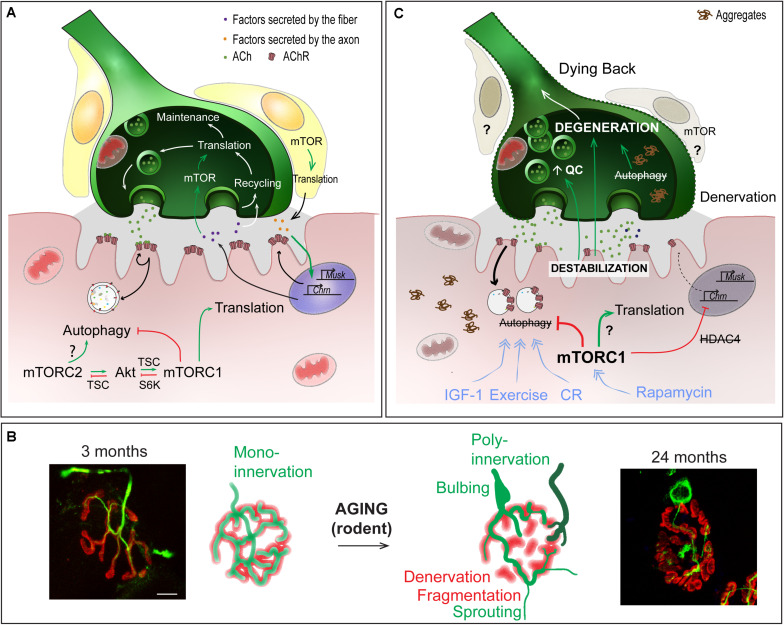
Role of mTOR-dependent processes in NMJ alteration upon aging. **(A)** Importance of protein synthesis and autophagy in the pre- and post-synaptic compartments of NMJs. **(B)** Age-dependent changes in NMJ morphology, showing post-synaptic changes (*e.g.* fragmentation, denervation) and compensatory changes in the pre-synaptic region (*e.g.*, sprouting, poly-innervation). Scale bar, 10 μm. **(C)** Pathomechanisms involved in NMJ deterioration in aged muscle, and associated with (m)TOR deregulation. Primary destabilization of the post-synaptic compartment likely involves autophagy blockade and defective turnover in synaptic proteins. Changes in neural transmission, including increase in quantal content (QC) may be an attempt to compensate for post-synaptic decline and may in turn further aggravate NMJ deterioration. Decline of the post-synaptic compartment and retrograde feedback from muscle onto motor neuron may lead to the degeneration of the motor axon (dying back process). Exercise, IGF1, rapamycin and caloric restriction (CR) may limit age-related NMJ deterioration by modulating autophagy and/or (m)TOR-associated complexes. Red lines indicate inhibition; Green arrows represent activation; blue arrows show the beneficial effect of treatments.

Several reports point to the role of local protein synthesis in long-term synaptic plasticity in the central nervous system for learning and memory (for a review, see Santini et al., 2014). Results obtained in both invertebrates and vertebrates suggest similar roles of local translation in the regulation of NMJ function. In crayfish, long-term facilitation occurs at NMJs of opener muscles from the leg in response to high frequency stimulation and requires local active translation (Beaumont et al., 2001). In *Drosophila* larvae, activation of PI3K in the motor neuron, upon glutamate release, would ensure the maintenance of neuronal activity, by promoting TOR/translation-dependent synaptic growth and, by limiting glutamate liberation *via* Akt-dependent inhibition of FOXO (Howlett et al., 2008). In rodents, studies on Spinal Muscular Atrophy (SMA) led to the identification of CTRP3 (*C1QTNF3, C1q/Tumor Necrosis Factor-related protein 3*), which is secreted by muscle and promotes protein synthesis locally in motor neurons, *via* the activation of mTOR (Figure 4A). In SMA, reduced levels of CTRP3 would limit protein synthesis and thereby, contribute to motor neuron degeneration (Rehorst et al., 2019).

#### Role of Local Translation in Muscle Sub-Synaptic Compartment

Early studies unveiled the accumulation of free ribosomes and endoplasmic reticulum in the sub-synaptic region of muscle fibers (Padykula and Gauthier, 1970). Moreover, free ribosomes accumulate underneath the sarcolemma shortly after denervation (Gauthier and Dunn, 1973; Gauthier and Schaeffer, 1974), which correlates with the synthesis of new AChRs and hypersensitivity of denervated muscle to acetylcholine (Figure 4A). In *Drosophila* larvae, aggregates containing eIF4E and PABP, together with polysomes, are observed in sub-synaptic regions of NMJs, consistent with local translation activity (Sigrist et al., 2000). It is noteworthy that there were no similar aggregates detected in the pre-synaptic regions. In mice, local inhibition of protein synthesis in the sub-synaptic region leads to rapid withdrawal of the motor axon and to the elimination of the NMJ (McCann et al., 2007). Consistent with a role of protein synthesis in the regulation of neuronal viability, depletion of the translation elongation factor eEF1A2 leads to motor axon degeneration, *via* a dying back process, in *Wst* mutant mice (Murray et al., 2008). In contrast, loss of eEF1A2 limits neuronal degeneration induced by nerve injury, suggesting that local translation at the NMJ differentially regulates dying back and Wallerian degenerative processes.

Beyond its role in NMJ maintenance, evidence has emerged for a role of translation in supporting NMJ plasticity. High neuronal activity triggers an increase in translation, especially of synaptic components, in sub-synaptic regions of *Drosophila* larval muscle, and thereby allows for pre- and post-synaptic remodeling (Sigrist et al., 2000; Menon et al., 2004). Interestingly, translation-mediated retrograde signaling from the post-synaptic compartment has been reported to control pre-synaptic activity in *Drosophila* larval muscle. Penney et al., first established that TOR-dependent translation in the post-synaptic region mediates the compensatory increase in neurotransmitter release in response to reduced post-synaptic activity (Petersen et al., 1997; Frank et al., 2006; Penney et al., 2012). TOR activation likely allows local translation of specific targets at the motor endplate, which act on the pre-synaptic compartment to adapt neuronal activity. It remains to be established how TOR becomes activated in the sub-synaptic muscle region and whether specific sub-synaptic transcripts are targeted for TOR-mediated translation. Whether adaptive or compensatory changes at NMJs in mammals involve similar local regulation of mTOR-dependent translation in the sub-synaptic muscle region also needs exploring.

### (m)TOR and the Regulation of Autophagy at the NMJ

#### Autophagy Regulation in Brief

Autophagy is an evolutionarily conserved homeostatic process that is induced under stressful or unfavorable conditions (Lum et al., 2005) but also ensures basal turnover of long-lived proteins and organelles. Autophagy proceeds through the formation of double membrane vesicles, called autophagosomes, that engulf large parts of cytoplasm and organelles to degrade them after fusion with lysosomes (Tanida, 2011; Figure 2D). In most cells, inhibition of (m)TORC1 is sufficient to induce autophagic flux, even in the presence of nutrients (Jung et al., 2010). The inhibitory effect of mTORC1 on autophagy is primarily mediated through regulation of the Ulk1/Atg13/FIP200 complex involved in autophagy induction (Chan, 2009; Hosokawa et al., 2009). mTORC1 directly phosphorylates Ulk1 and Atg13 and inhibits their activation by AMPK (Egan et al., 2011; Kim et al., 2011).

In skeletal muscle, a tight regulation of autophagy is essential to maintain homeostasis. Conditions like starvation, where mTORC1 activity is suppressed, strongly induce autophagy, which contributes to muscle atrophy (Mizushima et al., 2004). Genetic mutations or drugs that either induce or impair autophagic flux cause muscle atrophy and are associated with muscle degeneration or aggregate and vacuole accumulation, respectively (Wang et al., 2005; Masiero et al., 2009). Early studies suggested that autophagy in skeletal muscle was controlled by FOXO transcription factors independent of mTORC1 (Mordier et al., 2000; Mammucari et al., 2007; Zhao et al., 2007; Sandri, 2010; Yamazaki et al., 2010; Sanchez et al., 2012). However, in TSCmKO muscle (in which mTORC1 is always active), autophagic flux is blocked at the initiation step, because of the inhibitory phosphorylation of Ulk1. This occurs despite activation of FOXOs and high autophagy-related gene expression (Castets et al., 2013; Castets and Ruegg, 2013). Inversely, mTORC1 inactivation by raptor depletion increases autophagy induction, while reduced expression of FOXO-dependent autophagy genes may dampen degradation steps (Castets et al., 2013; Baraldo et al., 2020). Therefore, mTORC1 deregulation in muscle fibers strongly perturbs autophagy with deleterious consequences. In RImKO mice (*i.e.*, mTORC2 inactivation), changes in PKB/Akt phosphorylation (Ser473) do not affect the mTORC1 pathway and there are no signs of autophagy defects (Bentzinger et al., 2008; Castets et al., 2013), suggesting that mTORC2 is not involved in autophagy regulation in rodent muscle.

#### Importance of the Autophagic Flux in Pre- and Post-synaptic Compartments of NMJ

In *Drosophila* larvae, overexpression or knockdown of autophagy genes using specific neuronal drivers alters autophagy in motor neurons. An increase in autophagic flux promotes NMJ growth (Shen and Ganetzky, 2009). This is achieved by degrading specific factors, such as the E3 ubiquitin ligase Hiw, which, in turn, preserves factors involved in NMJ growth, such as Wnd (Shen and Ganetzky, 2009). Thus, the positive effects of rapamycin on NMJs reported in *Drosophila* may involve autophagy induction mediated by TORC1 inhibition (Knox et al., 2007; Shen and Ganetzky, 2009; Wong et al., 2015). Autophagy also contributes to the recycling of synaptic components, especially upon high frequency stimulation. Autophagy blockade in neurons hence leads to neurodegeneration associated with the accumulation of protein aggregates and inclusions (Hara et al., 2006; Komatsu et al., 2006, 2007; Soukup et al., 2016; Vanhauwaert et al., 2017).

At the motor endplate, the accumulation of synaptic proteins relies on their tightly regulated turnover, defined by the balance between synthesis and degradation. AChRs are degraded *via* selective autophagy, which involves the E3 ubiquitin ligase MuRF1 (TRIM63), the cargo protein p62, as well as endophilin B1, a SH3-domain protein involved in endocytosis (Rudolf et al., 2013; Khan et al., 2014). Upon denervation, high rates of autophagy-dependent AChR degradation, coupled with a marked up-regulation of AChR synthesis, lead to major increases in AChR turnover (Akaaboune et al., 1999; Bruneau and Akaaboune, 2006; Strack et al., 2011, 2015; Figure 4A). A recent study suggested that blocking autophagy may protect motor endplates, by limiting AChR degradation. Indeed, CGRP (α*-Calcitonin Gene-Related Peptide*) treatment induces mTORC1-dependent blockade of autophagy and limits endplate degeneration in denervated muscle (Machado et al., 2019). In contrast, several reports point to the need of functional autophagic flux for NMJ maintenance. In particular, muscle-specific deletion of *Atg7* blocks autophagy in muscle and causes spontaneous fiber denervation and increased fragmentation of motor endplates (Carnio et al., 2014). Consistent with autophagy blockade, AChRs accumulate in endocytic vesicles underneath the motor endplate (Khan et al., 2014). More surprisingly, AChR turnover is increased in Atg7-deficient muscle (Carnio et al., 2014), which suggests that their internalization and/or the incorporation of new receptors are augmented (Yampolsky et al., 2010; Strack et al., 2015). Whether this occurs in response to their abnormal accumulation in endocytic vesicles remains to be determined. As in Atg7-deficient muscle, endplate fragmentation in TSCmKO mice may arise from autophagy impairment, although AChR turnover is unchanged (Castets et al., 2019). Limited autophagy flux may also contribute to NMJ destabilization in muscle-specific, inducible Raptor-KO mice, as autophagy restoration with a Tat-Beclin1 peptide is sufficient to reduce fiber denervation (Baraldo et al., 2020). Further evaluation of AChR dynamics in Raptor-KO muscle may help confirm the role of autophagy in the observed motor endplate changes and rule out the possibility that Tat-Beclin1-mediated NMJ improvements arise from amelioration of the overall muscle phenotype. Together, these data support the notion that autophagy flux must be tightly regulated to maintain NMJ stability. It remains to be investigated as to how autophagy blockade leads to NMJ destabilization and whether/how it involves an abnormal increase in synaptic protein turnover. Moreover, whether specific blockade of autophagy (*e.g.*, in Atg7-deficient muscle) can preserve motor endplate stability in response to nerve injury and functionally support re-innervation capacity needs to be explored.

### Role of mTORC1 in the Transcriptional Regulation of Synaptic Genes

Constant mTORC1 activation in TSCmKO muscle precipitates denervation-induced motor endplate degeneration (Castets et al., 2019). In mutant mice, AChRs do not accumulate in endocytic vesicles, suggesting that the denervation-induced defect is not related to autophagy blockade. Instead, the failure of TSCmKO mice to stimulate AChR turnover upon denervation points to insufficient synthesis of new receptors, consistent with the defective up-regulation of the expression of genes encoding AChR subunits in TSCmKO denervated muscle (Castets et al., 2019). Interestingly, mTORC1-mediated dampening of PKB/Akt mediates the suppression of denervation-induced synaptic gene transcription through defective nuclear import (and hence activity) of HDAC4 (*Histone DeACetylase 4)* (Castets et al., 2019). HDAC4 contributes to denervation-induced synaptic gene expression by releasing HDAC9- and DACH2-dependent inhibition of the myogenic marker myogenin (Cohen et al., 2007, 2009; Tang et al., 2009; Moresi et al., 2010). Thus, the lack of incorporation of newly formed receptors at the post-synaptic plasma membrane is probably the reason for the destabilization of motor endplates in TSCmKO muscle upon denervation. It is unlikely that this contributes to endplate fragmentation in TSCmKO innervated muscle, as basal expression of synaptic genes is similar to that in controls. Hence, by controlling transcription and translation of synaptic genes, as well as autophagy-mediated degradation of synaptic proteins, PI3K/Akt/mTORC1 signaling is important for both stability and remodeling capacity of the motor endplate after injury.

### Role of mTOR in Schwann Cells

Several reports indicate a role of mTORC1 signaling in Schwann cell (SC)-mediated axon myelination in the peripheral nervous system (for a review, see Figlia et al., 2018). The role of SC mTOR activity in the development and maintenance of NMJs remains more elusive. In mice deficient for the PI3P-phosphatase PTEN (Phosphatase and TENsin homolog) and the GAP protein NF1 (Neurofibromin 1) in SCs, increased activity of Akt/mTORC1 delays the development of NMJs, with persistent immature AChR clusters, poly-innervation and reduced expression of synaptic genes at late embryonic stages (Li et al., 2019). Further investigations on the underlying mechanisms, especially on whether this implies changes in translation and/or autophagy in SCs and defective secretion of specific factors, such as agrin, by SCs, will be of interest.

Together, these data highlight that local changes in transcription, translation and autophagic degradation of synaptic components in pre- and post-synaptic compartments, as well as in SCs, are essential to maintain and orchestrate NMJ structure and function. Tight spatial and temporal control of TOR activity is required to facilitate these processes, but to fully understand the pathological effects of their perturbations in aging and disease, the intricate interplay between these cellular processes and TOR remains to be fully explored.

## mTOR as a New Therapeutic Target to Stabilize NMJs

mTORC1 signaling is deregulated in numerous pathologies, including cancer, where developing therapeutic inhibitors of the pathway is of major importance. Emerging evidence indicates that imbalanced mTORC1 signaling contributes to muscle atrophy in various neuromuscular diseases, as well as in aging. As muscle dysfunction severely affects the quality of life of patients and increases the risk of morbidity and mortality, efforts to counteract muscle deterioration is of major public health importance. Growing evidence suggests that manipulating mTOR in contexts such as aging may help to restore or preserve muscle homeostasis, in particular by its effect on NMJs.

### Role in Neurodegenerative Diseases – Amyotrophic Lateral Sclerosis

Neurodegenerative diseases are severe disorders affecting motor neurons, and consequently muscle. In the last decade, similarities in the pathomechanisms responsible for age-dependent nerve/muscle alterations and those responsible for neurodegenerative pathologies have emerged. In this section, we focus on the potential role of mTOR signaling in the pathology of Amyotrophic Lateral Sclerosis (ALS).

Amyotrophic Lateral Sclerosis is a fatal neurodegenerative disorder, caused by loss of motor neurons. Although most ALS cases are sporadic, genetic mutations in different genes, including *SOD1, FUS, C9ORF72*, or *UBQL2* are responsible for familial forms. Early reports suggested that the primary cause of ALS is the death of motor neurons. However, recent studies suggest that the primary pathologic events actually occur at NMJs or even in the muscle fibers themselves. A dying back process, starting from motor neuron terminals, would then cause retrograde degeneration of axons and neuron soma. In fly and rodent models of ALS, NMJ alterations and defective synaptic transmission precede the distal degeneration of motor neurons (Fischer et al., 2004; Rocha et al., 2013; Shahidullah et al., 2013; Tremblay et al., 2017; Chand et al., 2018; Martineau et al., 2018). Dynamic axonal sprouting and re-innervation have also been reported (Fischer et al., 2004; Martineau et al., 2018). Importantly, the expression of diseased forms of SOD1 (Superoxide Dismutase) in muscle leads to muscle atrophy, alters neuromuscular transmission and/or triggers the degeneration of motor neurons (Dobrowolny et al., 2008; Wong and Martin, 2010). Hence, it is possible that ALS primarily involves deleterious events in muscle fibers, which secondarily cause degeneration of the motor neuron, and in turn denervation of the muscle fibers.

Autophagy impairment plays a central role in ALS, causing protein aggregation in motor neurons and contributing to their death (Zhang et al., 2011). Consistent with autophagy blockade, Saxena et al. (2013) showed that mTORC1 activity increases in motor neurons in an ALS mouse model. However, mTORC1 activity seems to attenuate neuron degeneration since rapamycin precipitates the disease in mouse models of ALS (Zhang et al., 2011; Saxena et al., 2013). mTOR-dependent neuroprotection is associated with up-regulation of the transcriptional regulator, Btg2, which may promote neuroprotective pathways in motor neurons (Saxena et al., 2013). In contrast to rapamycin, treatment with trehalose is sufficient to increase lifespan of SOD1^*G*93*A*^ mice and limit motor neuron degeneration. Although there was no clear evidence of changes in autophagic flux, authors suggest that trehalose acts independently of mTORC1, by improving autophagy in late degradation stages (Zhang et al., 2014). One could hypothesize that reducing mTOR activation in motor neurons aggravates ALS, as it promotes autophagy induction in a context where the degradation steps are blocked. In contrast, drugs targeting later stages of autophagy may eventually prove beneficial. In parallel, it remains unclear whether autophagy is altered in muscle fibers, especially in sub-synaptic regions, and whether this contributes to primary NMJ destabilization. Heightened AChR turnover, related to high autophagy flux, has been suggested to contribute to endplate deterioration in SOD^*G*93*A*^ mice. However, these changes were ascribed to PKC deregulation rather than mTORC1 (Dobrowolny et al., 2018). Still, local IGF-1 expression in muscle of ALS mice reduces motor neuron degeneration and NMJ deterioration (Dobrowolny et al., 2005). This effect may involve a combination of mTORC1-dependent anabolic processes and mTORC1-independent increases in autophagy within motor neurons. Interestingly, a recent report unveiled down-regulation of AChR expression in sub-synaptic myonuclei mediated by the ALS-associated mutant FUS protein (Picchiarelli et al., 2019). Therefore, defective synaptic gene regulation in muscle may contribute to the primary events triggering NMJ destabilization in ALS. Further investigations focusing on the potent effects of mTOR on synaptic gene deregulation in sub-synaptic muscle regions and the capacity of mTOR-targeting strategies to counteract these effects will be of major interest.

### Role of mTOR in Sarcopenia and Effects of mTOR Targeting Strategies

Preserving muscle quality in aging has become in the last years a worldwide public health issue with major clinical, social and economic impacts. Both mTORC1 and the NMJ are strongly implicated in the progression of age-related muscle loss, or sarcopenia. In the following, we focus on the potential role of mTOR signaling in age-related NMJ deterioration and on strategies targeting mTOR to limit these defects.

#### Defects in NMJs in Sarcopenia

One hallmark of sarcopenia is deterioration of NMJs and muscle fiber innervation. The sequence of events leading to NMJ deterioration remains debated, particularly in regards to whether changes in the motor axon or muscle fiber initiate these events. Regardless of the primary cause, NMJ dysfunction is thought to play a central role in the age-related loss of muscle function. Evidence is, however, lacking regarding its role in initiating this deterioration. Multiple morphological and functional changes occur in aging NMJs (Figures 4B,C). Denervated muscle fibers become more prevalent in muscle from both aged humans and rodents (Deschenes et al., 2010; Valdez et al., 2010; Chai et al., 2011; Chung et al., 2017; Sheth et al., 2018). Together with denervation, motor endplate area shrinks and post-synaptic AChR clusters become fragmented with age (Banker et al., 1983; Valdez et al., 2010; Cheng et al., 2013). However, growing evidence refutes a direct role of motor endplate fragmentation on NMJ function or sarcopenic progression (Willadt et al., 2016; Jones et al., 2017). Similarly, abnormal pre-synaptic patterns, such as poly-innervation and sprouting (Arizono et al., 1984; Oda, 1984; Wokke et al., 1990; Valdez et al., 2010; Cheng et al., 2013) are now thought to be compensatory processes aimed at re-establishing innervation of fibers left denervated following motor unit loss, rather than detrimental factors. Nevertheless, fragmentation, poly-innervation and sprouting are all indicative of either synaptic remodeling or instability. In fact, defects in sprouting may actually be responsible for exacerbating muscle fiber denervation and loss of muscle function at high age (Arizono et al., 1984). Several, sometimes contradictory, reports point to changes in the motor neuron terminal organization and in neurotransmission (Gutmann et al., 1971; Banker et al., 1983; Smith, 1984; Chen et al., 2012; Mahoney et al., 2014). These pre-synaptic changes may actually well be the attempts to compensate for post-synaptic decline (Courtney and Steinbach, 1981; Arizono et al., 1984; Khosa et al., 2019), and may arise from retrograde feedback from muscle onto the motor neuron (Ouanounou et al., 2016). Hence, destabilization of the post-synaptic compartment is likely a primary event in the progressive loss of integrity of NMJs and muscle, and may in particular lead to the degeneration of the motor neuron in a dying back process (Chai et al., 2011; Chung et al., 2017; Sheth et al., 2018).

#### Changes in mTORC1 Signaling With Age and Role in Sarcopenia-Associated NMJ Defects

Muscle atrophy is usually associated with an imbalance between protein synthesis and protein degradation. First reports on the metabolic capacity of sarcopenic muscles suggested that anabolic pathways, such as Akt/mTORC1, are less active in muscle from aged individuals, as compared to young ones. Indeed, sarcopenic muscle shows impaired or delayed response to anabolic stimuli, such as amino acids, IGF-1 injection, exercise or electric stimulation [“anabolic resistance” – (Guillet et al., 2004; Parkington et al., 2004; Cuthbertson et al., 2005; Funai et al., 2006; Kumar et al., 2009; Fry et al., 2011; Burd et al., 2013; Barclay et al., 2019)]. However, several studies also point to higher basal mTORC1 activation in aged or sarcopenic rodent muscle (Barns et al., 2014; Baar et al., 2016; White et al., 2016; Joseph et al., 2019; Tang et al., 2019; Ham et al., in press) and in humans (Markofski et al., 2015), which may mediate the blunted response to nutrition and exercise. The up-stream factors leading to mTORC1 activation with aging remain elusive. In fact, lower levels of IGF-1 in the circulation (Lamberts et al., 1997; Leger et al., 2008; Hofmann et al., 2015) together with local changes associated with inflammation are thought to contribute to age-related muscle decline (Owino et al., 2001; Hameed et al., 2002; Barbieri et al., 2003). Indeed, increasing muscle IGF-1 levels effectively counteracts age-related muscle atrophy and NMJ alterations in mice (Barton-Davis et al., 1998; Messi and Delbono, 2003; Payne et al., 2007; Ascenzi et al., 2019). Interestingly, only mild age-related changes in PKB/Akt activation have been reported (Clavel et al., 2006; Leger et al., 2008; White et al., 2016). The apparent discrepancy between low IGF-1 signaling and high mTORC1 activation in aged muscle suggests an independent contribution of IGF-1 and mTORC1 signaling in sarcopenia. As nerve injury strongly activates mTORC1, it is plausible that denervation in sarcopenic muscle precedes and contributes to activation of mTORC1, which may, in turn, further disrupt NMJs.

In line with heightened mTORC1 activity, evidence that autophagy impairment contributes to muscle aging is also emerging (Figure 4C). In *Drosophila*, decreased autophagic flux precipitates abnormal protein aggregate accumulation, while improving autophagic flux slows age-related muscle deterioration (Demontis and Perrimon, 2010). Signs of dampened autophagic flux have also been observed in aging rodent and human muscle, including abnormal organelle and protein aggregate accumulation and reduced autophagy marker expression (Drummond et al., 2008; Wohlgemuth et al., 2010; Kim et al., 2013; Sakuma et al., 2016; Sebastian et al., 2016; White et al., 2016). In Atg7-deficient muscle, autophagy blockade accelerates the appearance of age-related NMJ alterations (Carnio et al., 2014). Interestingly, reduced IGF-1 levels may actually contribute to sarcopenia, by limiting autophagic flux *via* mTORC2 and PKC (Renna et al., 2013; Ascenzi et al., 2019). As previously mentioned, autophagy in motor neuron terminals is also important to sustain protein turnover associated with synaptic vesicle cycling. Therefore, defective autophagy in motor neurons may progressively impinge normal axonal trafficking and synaptic function with age, ultimately leading to motor neuron death and muscle fiber denervation. Several other factors, including reactive oxygen species, mitochondrial defects, satellite cell dysfunction and low grade inflammation, also contribute to NMJ destabilization and may also be influenced by high mTOR signaling in aging muscle (for a review, see Ham and Rüegg, 2018).

#### Strategies Targeting mTOR to Slow-Down NMJ Alterations Upon Aging

Based on the low anabolic response to amino acids in sarcopenic muscle and low protein intake in some elderly populations, amino acid supplementation and/or improved dietary habits are frequently recommended as strategies to limit sarcopenia (Drummond et al., 2008; Robinson et al., 2012). In contrast, long-term caloric restriction slows muscle aging and preserves both pre- and post-synaptic structures of the NMJ (Valdez et al., 2010). Caloric restriction may restore autophagic flux in sarcopenic muscle by suppressing mTORC1 (Valdez et al., 2010; Wohlgemuth et al., 2010). Similarly, exercise, which may reduce anabolic resistance and release autophagy inhibition in sarcopenic muscle (He et al., 2012; Kim et al., 2013; Luo et al., 2013; Lenhare et al., 2017), reduces both pre-synaptic changes (*e.g.*, reduction in quantal content) and post-synaptic muscle abnormalities with aging (Fahim, 1997; Valdez et al., 2010; Cheng et al., 2013). As discussed above, treatment with IGF-1 or analogous drugs is also sufficient to counteract age-related muscle loss and NMJ deterioration. As in ALS, this may involve mTORC1-dependent anabolic processes, mTORC2-dependent autophagy induction, and improved satellite cell function (Barton-Davis et al., 1998; Chakravarthy et al., 2000; Messi and Delbono, 2003; Payne et al., 2007; Ascenzi et al., 2019; Pennuto et al., 2020). Although IGF-1 treatment seems promising, the risk of cancer, associated with its anti-apoptotic effect, still raises concerns. Surprisingly, inhibition of mTORC1 by rapamycin or rapalogs is also sufficient to reverse age-associated muscle alterations. In particular, rapamycin/rapalogs limit NMJ alterations and restore autophagic flux (Joseph et al., 2019; Tang et al., 2019; Ham et al., in press). Although consistent with the overall beneficial impact of rapamycin on organismal aging, further investigation focusing on the distinct effects of these drugs on different muscles and on the consequences of inhibiting mTORC1 signaling pathway on muscle mass and function will be essential. Together, these data suggest that NMJ destabilization in both ALS and sarcopenia primarily involves post-synaptic changes, followed by adaptive changes in the pre- and post-synaptic compartments, which precede motor neuron degeneration. The role of local mTORC1 activity in initiating and/or accentuating these changes remains to be established. With it, efforts to identify the mechanisms triggering changes in TOR signaling and solutions to maintain the delicate balance between protein synthesis and degradation at the NMJ are essential.

## Conclusion

Growing evidence points to a prominent role of TOR signaling in the formation and maintenance of the NMJ. While most *Drosophila* studies have implied an important function of TOR signaling in the pre-synaptic compartment, work in mice has demonstrated the importance of tightly controlled mTOR signaling in the post-synaptic compartment. The different focus on pre- and post-synaptic compartments between species may result from several factors, including the distinct nature of the chemical synapses (*i.e.*, glutamatergic *vs.* cholinergic), different NMJ organization and developmental mechanisms, stage of developmental analysis (*i.e.*, developing vs. adult), and/or technical limitations relating to modulating factors specifically in pre- and post- synaptic compartments. Interestingly, in *Drosophila*, evidence suggests that TORC1 and TORC2 play in concert to regulate NMJ development and plasticity. In contrast, mTORC1 in rodents is predominantly involved in the formation and maintenance of the NMJ. Perturbations in mTORC1 activity likely contribute or even initiate NMJ destabilization in pathological conditions, by altering the finely tuned balance between synthesis (transcription and translation) and degradation (*via* autophagy and proteasome) of synaptic components. However, future investigations should aim to delineate the effects of TOR deregulation on muscle homeostasis from those on NMJ maintenance, by characterizing the consequences of local changes in TOR activity at the endplate or in the terminal branch of the motor neuron. Similarly, efforts to identify the primary mechanisms leading to TOR deregulation in pathological conditions involving NMJ/muscle affection, such as sarcopenia or ALS, need to be made. Further understanding of the complex mechanisms involving (m)TOR signaling in NMJ physiology will uncover essential insights for the design of therapeutic strategies to counteract or limit the loss of NMJ integrity and muscle function in aging and neurodegenerative disorders.

## Author Contributions

PC wrote and edited the manuscript. DH and MR edited the manuscript. All authors contributed to the article and approved the submitted version.

## Conflict of Interest

The authors declare that the research was conducted in the absence of any commercial or financial relationships that could be construed as a potential conflict of interest.

The reviewer RR declared a past co-authorship with several of the authors PC, MR to the handling editor.
